# The Evaluation of Teleost-Derived Antimicrobial Peptides Against Neisseria gonorrhoeae

**DOI:** 10.7759/cureus.57168

**Published:** 2024-03-29

**Authors:** Po-Wei Huang, Chung-Yi Liou, Ying-Chen Lee, Tzu-Yu Wei, Han-Chen Ho, Tsung-Ying Yang, Liang-Chun Wang

**Affiliations:** 1 Department of Marine Biotechnology and Resources, National Sun Yat-Sen University, Kaohsiung, TWN; 2 Division of Urology, Department of Surgery, Zuoying Armed Forces General Hospital, Kaohsiung, TWN; 3 Division of Urology, Department of Surgery, Tri-Service General Hospital, National Defense Medical Center, Kaohsiung, TWN; 4 Center of General Education, Shu-Zen Junior College of Medicine and Management, Kaohsiung, TWN; 5 Department of Anatomy, Tzu Chi University, Hualien, TWN; 6 Department of Medical Laboratory Science, I-Shou University, Kaohsiung, TWN

**Keywords:** infection, biofilm, antimicrobial peptides, gonorrhea, sexually transmitted infection

## Abstract

Introduction

Gonorrhea has become an emerging sexually transmitted infection worldwide. The multi-antibiotic resistance facilitates the transmission; thus, new antibiotics or alternatives are needed. Antimicrobial peptides (AMP) are antimicrobials naturally secreted by the host as a defense material. Teleost-derived AMP have gained attention over the past two decades due to their potent efficacy toward microorganisms. This study examines teleost-derived AMP against *Neisseria gonorrhoeae *(GC), the responsible bacteria for gonorrhea, to evaluate the antibiotic potential as a future alternative for preventing gonorrhea.

Methods

Minimal inhibitory concentration (MIC) and time-killed assay were conducted to evaluate the inhibition concentration of each AMP. Transmission electron microscopy was used to confirm the potential mode of action. The inhibition of microcolony formation and adherence to epithelial cells were examined to assess the infection inhibition.

Results

Pardaxin-based (flatfish pardaxin {PB2}) and piscidin-based (striped bass piscidin 1 {PIS} and tilapia piscidin {TP} 4) AMP were effective toward GC under or equal to 7.5 μg/mL as of minimal inhibitory concentration. Transmission electron microscopy images revealed that these AMP attack bacterial membranes as membrane blebbing and breakage were observed. These AMP also effectively reduced the GC biofilm formation, as well as their adherence to human endocervical epithelial cells.

Conclusion

Pardaxin-based (PB2) and piscidin-based (PIS and TP4) teleost-derived AMP can inhibit GC and potentially serve as the new antibiotic alternative for preventing GC colonization and infection. This study will shed some light on the future development of teleost-derived AMP in treating gonorrhea and maintaining reproductive health.

## Introduction

Gonorrhea is one of the most common sexually transmitted infections and is caused by the human-obligated bacterium *Neisseria gonorrhoeae* (GC) [1]. The infection can affect both males and females in their young and early adulthood. Notably, the females can develop serious complications, such as pelvic inflammatory disease and disseminated gonococcal infection, which may lead to ectopic pregnancy or even infertility [2]. In addition, asymptomatic infections were reported as up to 56% and 80% in males and females, respectively [3]. Along with the increasing antibiotic resistance, GC infection has been a threat to reproductive health, as well as a burden to the healthcare system.

Antibiotic-resistant gonorrhea is seen as a significant public health issue worldwide that increases both social and economic costs [4]. The current treatment strategy was given as two antibiotic options: ceftriaxone and/or azithromycin, even though increasing rates of ceftriaxone and/or azithromycin resistance were reported in several countries [5]. New antibiotics, including macrolide-based solithromycin and spiropyrimidinetrione-based zoliflodacin, are under clinical trial [6,7]. The early development of SMT-571 and DIS-73285 [8,9] has also shown potential anti-GC effects. However, the current gonorrhea infection with antibiotic resistance still highlights the urgent requirement for new-class antibiotics and/or alternatives.

Antimicrobial peptides (AMP), secreting from various tissues in vertebrates and invertebrates, have shown broad-spectrum activity against microorganisms and have been considered promising alternatives for antibiotics [10]. AMP are mostly positively charged and amphiphilic, essential for interacting with negatively charged bacterial membranes [11]. Due to potential selectivity against bacterial membranes [12], AMP are potentially active against a large spectrum of bacteria at nontoxic concentrations for mammalian cells. Moreover, because AMP targets the microorganisms' membrane, this may reduce antibiotic resistance development as other antibiotics target enzymatic activities or receptor-binding processes. In the past two decades, hundreds of cationic antimicrobial peptides have been identified from diverse animal sources [13]. To date, only a few studies have reported AMP's efficacy on GC, including human defensin 2, rabbit defensin 2, porcine protegrin-1, horseshoe crab tachyplesin-1, human LL-37, and amphibian dermaseptin [14,15]. A recent study of peptides against the functional unit of an efflux pump, which can export numerous antibiotics, has also been a novel research direction but limited to the combinational use with antibiotics [16]. Teleost-derived AMP, including hepcidin, β-defensins, cathelicidins, and piscidins, are of interest due to their potency against a broad spectrum of pathogenic bacteria [17]. Hence, this study explores the efficacy of teleost-derived AMP, including the pardaxin, hepcidin, and piscidin families, against GC. Moreover, infection-associated colonization and biofilm formation are also examined. The information from this study would help the future development of clinical prevention or treatment for the rising multidrug-resistant gonorrhea.

## Materials and methods

Bacterial strains

The *Neisseria gonorrhoeae* MS11 strain was kindly provided by Dr. Daniel C. Stein from the University of Maryland, College Park. The WHO gonococcal reference strain Z (WHO Z) was purchased from the National Collection of Type Cultures (NCTC), London, United Kingdom. GC was grown on GCK agar (Difco, BD Biosciences, Franklin Lakes, NJ) supplemented with 1% Kellogg's nutrient supplement at 37°C with 5% carbon dioxide (CO_2_) for 16-18 hours before use. Pili-positive colonies used in the experiments were identified using a dissecting light microscope based on colony morphology.

Epithelial cells

Human ME180 endocervical epithelial cells (American Type Culture Collection {ATCC} number HTB-33) were purchased directly from ATCC and maintained in RPMI 1640 (Sigma-Aldrich, St. Louis, MO) supplemented with 10% heat-inactivated fetal bovine serum (FBS) and penicillin/streptomycin mixture (100 unit penicillin and 0.1 mg streptomycin/mL, Sigma-Aldrich) at 37°C with 5% CO_2_ in saturated humidity. Cells were seeded onto 96 tissue-culture-treated plates (Thermo Fischer Scientific, Waltham, MA) and cultured for 24-48 hours depending on cell confluence. The culture medium was replaced with an antibiotic-free medium the day before the experiment.

Antibiotic susceptibility testing

The antibiotic susceptibilities of GC, including ciprofloxacin, azithromycin, and ceftriaxone, were determined using a disk diffusion test (Becton Dickinson Caribe, Ltd., Franklin Lakes, NJ) in accordance with the guideline recommended by the Clinical and Laboratory Standards Institute (CLSI) [18]. Briefly, the overnight colonies (16-20 hours) were suspended and diluted in GCP broth to a concentration of optical density (OD) = 0.1 at 650 nm. The bacterial suspension was then plated onto GC agar plates using swabs, and the disks were placed on the bacterial lawn with sterile tweezers. The plates were incubated at 37°C with 5% CO_2_ for 24 hours. The diameter of the inhibition zone was measured to determine the susceptibility according to the criteria recommended by the CLSI standard.

Minimal inhibitory concentration (MIC)

The synthetic AMP (Table 1) were purchased (Mission Bio, Taipei, Taiwan), and the inhibition activity test against GC was adapted from Bergman et al. [19]. Briefly, GC were suspended in GCP broth to a concentration of 105 colony-forming units (CFU)/mL, and 180 μL of this suspension was added to each well of a 96-well plate with 20 μL peptide solution at various concentrations. The final concentration of AMP in each well ranged from 0.08 to 20 μg/mL. The samples were incubated at 37°C with 5% CO_2_ for 24 hours. After the incubation, 10 μL of the suspension from each well was spotted on GCK agar plates. The MIC was determined as the lowest concentration of AMP that could reduce at least 90% of the bacteria relative to the non-AMP control.

**Table 1:** Details and properties of antimicrobial peptides used in this study [20-22]. The predicted properties, including the isoelectric point (pI) and Boman index listed above, were generated using the Antimicrobial Peptide Calculator and Predictor by the University of Nebraska Medical Center (Omaha, NE) (https://aps.unmc.edu/prediction). MW, molecular weight; PB2, flatfish pardaxin; PIS, striped bass piscidin 1; TP, tilapia piscidin; TH, tilapia hepcidin

AMP	Amino acid sequence	Family	Length	MW	pI	Boman index
PB2	GFFALIPKIISSPLFKTLLSAVGSALSSSGGQE	Pardaxin	33	3323.88	8.59	-0.35
TH1-5	GIKCRFCCGCCTPGICGVCCRF	Hepcidin	22	2329.91	8.54	0.18
PIS	FFHHIFRGIVHVGKTIHRLVTG	Piscidin	22	2572.06	12.01	0.7
TP3	FIHHIIGGLFSVGKHIHSLIHGH	Piscidin	23	2557.0	8.79	-0.34
TP4	FIHHIIGGLFSAGKAIHRLIRRRRR	Piscidin	25	2981.6	12.70	2.62

Time-kill assay

Time-kill curve analyses were conducted following the procedures adapted from Foerster et al. [23]. GC was cultured in GCP broth, supplemented with 1% Kellogg's supplement and 4.2% sodium bicarbonate (NaHCO_3_), in the presence of AMP concentrations that were serially diluted, encompassing values below and above the MIC. For each strain, 30 μL of the inoculum containing 108 CFU/mL was diluted in 15 mL of pre-warmed supplemented GCP. Subsequently, 90 μL of this mixture was dispensed into the round-bottom wells of 96-well microtiter plates. To each well containing 90 μL of pre-incubated bacteria, 10 μL of one of the antimicrobial concentrations was added. The plates were pre-incubated for four hours with agitation at 550 revolutions per minute (rpm) and 36°C in a humid PST-60HL thermo-shaker (Biosan, Riga, Latvia). After the preincubation, 10 μL of the GC suspension from each well was spotted onto GCK agar plates and incubated overnight. This allowed us to determine the viable count of GC.

Inhibition of microcolony formation

GC was suspended in GC broth media supplemented with Kellogg's supplement. The suspension was then diluted to a concentration of 107 CFU/mL. Two hundred microliters of GC were incubated in eight-well coverslip-bottom chambers (Sigma-Aldrich, St. Louis, MO) at 37°C with 5% CO_2_ for a static incubation period of four hours. Microscopic images were captured using a ZEISS Axio Observer light microscope (Oberkochen, Germany). The size of the GC microcolony within each aggregate of more than six individual bacterial cells was measured using National Institutes of Health (NIH) ImageJ (Bethesda, MD).

Cytotoxicity assay

ME180 cells were seeded into 96-well microtiter plates at a 5 × 10^3^ cells/well density and incubated for 24 hours. A final concentration of 10 μg/mL of AMP and 0.1% of positive control Triton-X were added to the well and incubated for 24 hours. Next, the Cell Counting Kit-8 (CCK-8) solution (Tools Biotech Inc., New Taipei City, Taiwan) was added and incubated for one hour. The absorbance at 450 nm was measured in each well, and the cytotoxicity level was analyzed according to the manufacturer's instructions.

Adherence assay

ME180 cells were incubated with GC (106 CFU/well) in the absence or presence of 10 μg/mL of tested AMP at 37°C and 5% CO_2_ for three hours. Cells were then lysed with 1% saponin in sterile phosphate-buffered saline (PBS) and spread onto the GCK agar plate. The number of adherent GC was counted to determine the effect of AMP.

Transmission electron microscopy (TEM)

GC with or without AMP treatment was centrifuged into pellets. Pelleted specimens were initially fixed in 2.5% glutaraldehyde/0.1 M sodium cacodylate buffer (pH 7.3) containing 1% tannic acid at 4°C overnight. After rinsing in 0.1 M sodium cacodylate buffer with 5% sucrose, specimens were then postfixed with 1% osmium tetroxide in 0.1 M sodium cacodylate buffer at room temperature for two hours. Following this, specimens were rewashed in buffer, stained with 2% aqueous uranyl acetate, and dehydrated through a graded series of ethanol followed by two washes in 100% acetone. The specimens were further infiltrated and embedded in Spurr's resin. Ultrathin sections, approximately 70 nm thick, were then sliced using a diamond knife on a Leica Ultracut R Ultramicrotome (Leica Geosystems, Heerbrugg, Switzerland) and observed with a Hitachi H-7500 transmission electron microscope (Hitachi, Tokyo, Japan) at 80 kV. Images were captured using an Advanced Microscopy Techniques (AMT) NanoSprint12 camera system (Advanced Microscopy Techniques, Woburn, MA).

Statistical analysis

Statistical significance was evaluated using one-way analysis of variance (ANOVA) followed by post hoc Dunnett's test when applicable. Prior to analysis, all data were assessed for normality using the Shapiro-Wilk test to ensure they followed a Gaussian distribution. The statistical analyses were conducted using GraphPad Prism 10 software (GraphPad Software, San Diego, CA).

## Results

Minimum inhibitory concentration

To examine the MIC of AMP (Table 1), a broth dilution assay was used to determine the growth inhibition of two GC strains: MS11 and WHO Z. The concentrations of each AMP were determined based on dermaseptin [15] and thus range from one to 20 μg/mL. Growth inhibition was observed in MS11 with flatfish pardaxin (PB2), striped bass piscidin 1 (PIS), and tilapia piscidin (TP) 4 but not with tilapia hepcidin (TH) 1-5 and TP3 (Table 2), with the MIC of PB2, PIS, and TP4 at 5, 7.5, and 7.5 μg/mL, respectively. Additionally, the MIC of the three AMP against WHO Z increased to 20 μg/mL for PB2 and 10 μg/mL for PIS and TP4. The data suggested that teleost-derived AMP were effective toward GC, but their efficacy depends on the type of AMP and GC strains.

**Table 2:** Antibiotic susceptibility and minimal inhibitory concentration (MIC) of AMP in MS11 and WHO Z strain. The susceptibility was determined by the disk diffusion method according to CLSI criteria. The MIC was determined as the lowest concentration of AMP that reduced equal to or more than 90% of bacterial growth compared to the controls. Results are representative of three independent experiments and shown as means. R, resistant; I, intermediate; S, susceptible; AMP, antimicrobial peptides; CLSI, Clinical and Laboratory Standards Institute; PB2, flatfish pardaxin; PIS, striped bass piscidin 1; TP, tilapia piscidin; TH, tilapia hepcidin

Strain	Antibiotic susceptibility			MIC of AMP (μg/mL)				
	Ceftriaxone	Ciprofloxacin	Azithromycin	PB2	TH1-5	PIS	TP3	TP4
MS11	S	S	S	7.5	>10	5	>10	7.5
WHO Z	I	R	R	20	>20	10	>20	10

Time-kill curve

**Figure 1:**
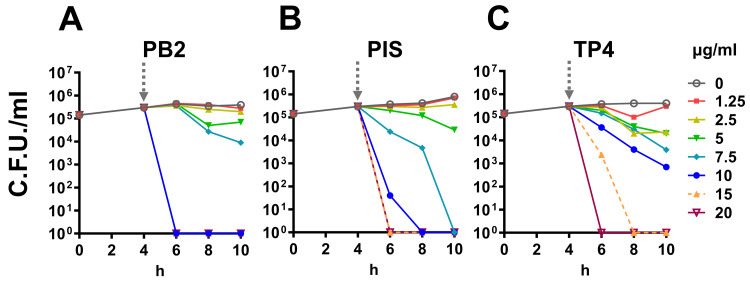
Time-kill curve of PB2, PIS, and TP4. The MS11 were seeded into a 96-well plate and incubated for four hours. (A) PB2, (B) PIS, or (C) TP4 was then added to the well after four-hour incubation. Eight to 11 doubling dilutions are plotted in different colors, and growth in the absence of antimicrobial was plotted in red. The AMP was added at timepoint four hours (dashed arrow) and monitored until timepoint 10 hours. The dots represented the means of each group. All experiments were conducted in triplicates. CFU, colony-forming units; AMP, antimicrobial peptides; PB2, flatfish pardaxin; PIS, striped bass piscidin 1; TP4, tilapia piscidin 4

To investigate the efficacy of AMP and mode of action through time-dependent manner, a time-kill assay was conducted. Serial concentrations of PIS, PB2, and TP4 were added into four-hour broth-cultured MS11 suspension. The suspension was then incubated and plated on GCK agar with serial dilution every two hours for a total of six-hour period. GC growth inhibition was determined by colony count compared to non-treatment control. We found that PIS and TP4 at equal and above 5 μg/mL continuously inhibited GC growth whereas PB2 at equal and above 7.5 μg/mL (Figure 1). Moreover, we observed that the inhibition curve over time in PB2 (Figure 1A) represented threshold-based compared to PIS and TP4 (Figure 1B, 1C), which were dose-dependent. These data indicated that all three AMP used here possess bactericidal properties, though the actual mode of action might differ between pardaxin- and piscidin-based AMP against GC.

Cellular effects of PB2, PIS, and TP4 on GC

**Figure 2:**
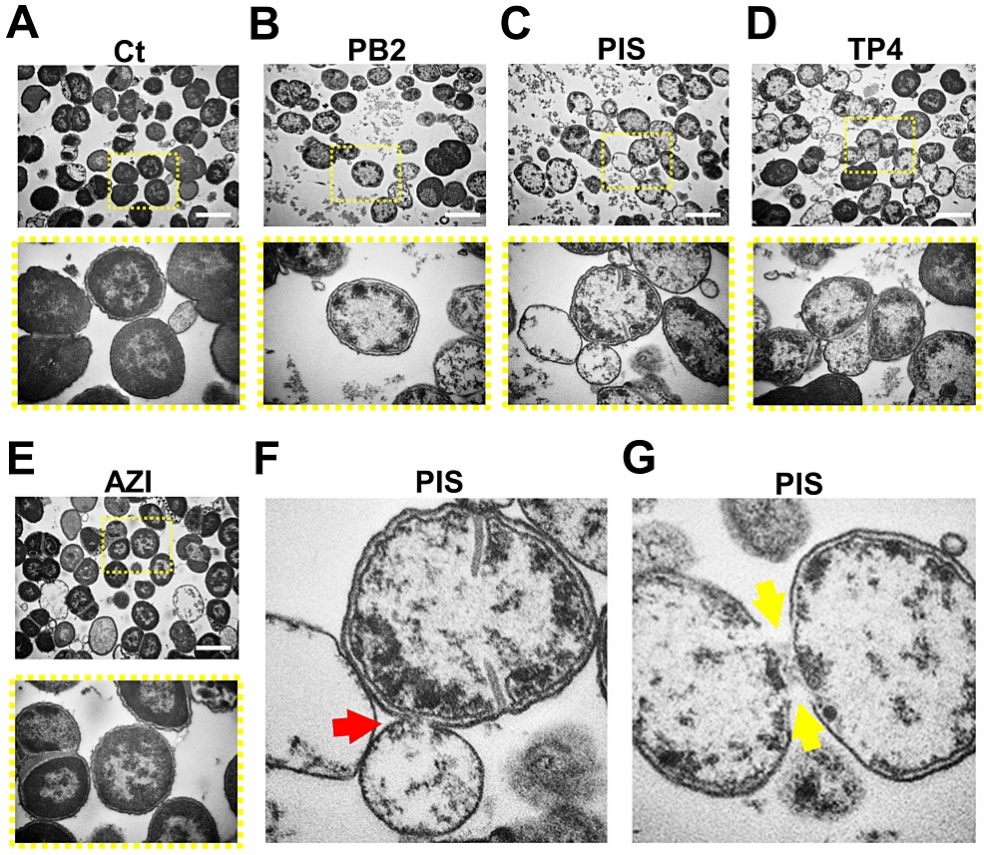
Morphological and structural impact of PB2, PIS, and TP4 on GC. Freshly grown MS11 were incubated in GCP broth in the absence or presence of AMP (10 μg/mL) or azithromycin (AZI) (0.5 μg/mL) for four hours. Cells were fixed and processed for TEM. Representative images of (A) non-treated GC (Ct), (B-D) AMP-treated GC, and (E) azithromycin-treated GC with low (upper panels) and high (lower panels) magnifications are shown. Notable phenotypic changes, such as membrane (F) blebbing and (G) breakage, were observed in all AMP-treated groups. Scale bar: 1 μm. Yellow dashed rectangles highlight the representative area. Red arrow, GC with membrane blebbing; yellow arrow, GC with membrane breakage. AMP, antimicrobial peptides; TEM, transmission electron microscopy; Ct, control; PB2, flatfish pardaxin; PIS, striped bass piscidin 1; TP4, tilapia piscidin 4

The teleost-derived AMP used here were all reported to have lipophilic properties by inserting and disrupting bacterial cell membranes for their mode of action [17]. To confirm the mode of action of PB2, PIS, and TP4, we treated MS11 with 10 μg/mL of each AMP for four hours and examined the cell morphology and structure under a transmission electron microscope. Azithromycin, a non-membrane-associated antibiotic, was used as a control. The non-treatment control showed a typical cocci shape (Figure 2A), double-membrane structure, and electron-dense cytoplasmic contents, whereas AMP-treated GC overall represented membrane blebbing, breakage, and deformation with electron-lucent cytoplasm and electron-dense particles accumulated near the inner membrane (Figure 2B-2D). In contrast, azithromycin-treated GC represented only the typical toroidal nucleoid with dense content in a cell and intact membranes (Figure 2E).

**Figure 3:**
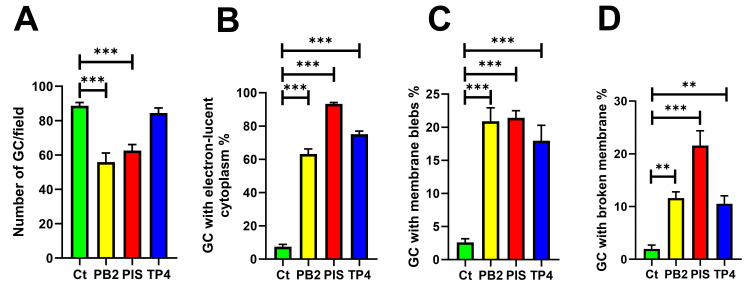
Quantification of morphological and structural changes of GC under PB2, PIS, and TP4 treatment. The cell number was quantified and shown as (A) the number of GC per field. Structural and morphological changes in cells, including (B) GC with electron-lucent cytoplasm, (C) membrane blebs, and (D) broken membrane, were quantified and shown as the percentage of GC within the non-treatment control (Ct). Data points represent individual randomly acquired eight TEM images. The bars represented the means of each group with standard deviations. Statistical significance was determined using a one-way ANOVA followed by post hoc Dunnett's test. ***p < 0.001 **p < 0.01 TEM, transmission electron microscopy; ANOVA, analysis of variance; PB2, flatfish pardaxin; PIS, striped bass piscidin 1; TP4, tilapia piscidin 4

Since a notable cell number reduction was found in the micrographs of the AMP-treated groups, the cell number per field was quantified, with the significantly reduced number of GC cells in PB2- and PIS-treated groups (Figure 3A). The membrane damages, such as electron-lucent cytoplasm, membrane blebbing, and breakage, were also counted for those different from untreated cells, with the most significant damage found in PIS-treated GC (Figure 3B-3D). The results suggested that PB2, PIS, and TP4 may share similar membrane-based antimicrobial effects but at different levels of damaging GC, therefore confirming the bactericidal property by targeting the bacterial membrane.

PB2, PIS, and TP4 reduced the formation of GC microcolony

**Figure 4:**
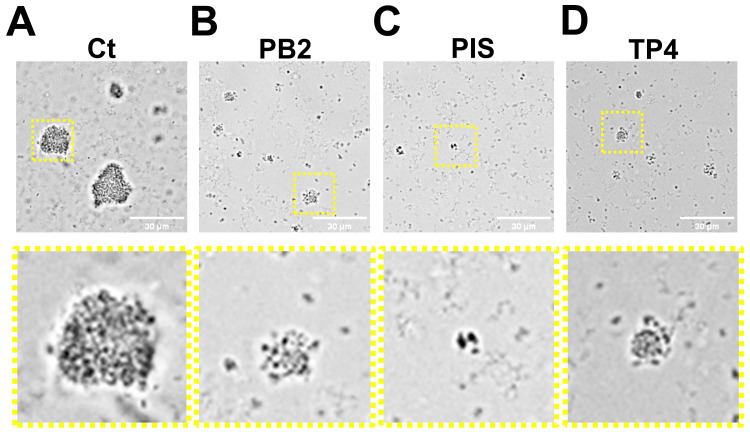
Effects of PB2, PIS, and TP4 on GC microcolony formation. MS11 were seeded in a coverslip-bottom chamber in the presence or absence of designated AMP, incubated for four hours and imaged by ZEISS Axio Observer light microscope. Representative images of (A) non-treated GC (Ct) and (B-D) AMP-treated GC are shown. Scale bar: 30 μm. All experiments were conducted at least in triplicates. AMP, antimicrobial peptides; Ct, control; PB2, flatfish pardaxin; PIS, striped bass piscidin 1; TP4, tilapia piscidin 4

**Figure 5:**
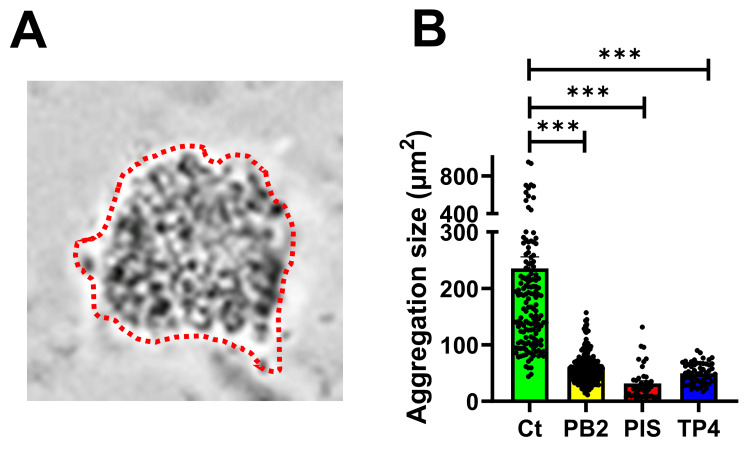
Quantification of the size of GC microcolony under PB2, PIS, and TP4 treatment. (A) The sizes of aggregations were evaluated by the average occupying area of individual aggregates in each image using NIH ImageJ (red dashed line). (B) The size of non-treated (Ct) and AMP-treated GC was quantified. The data were generated from 10 randomly acquired fields from four independent experiments. Each data point indicates an individual aggregation. The bars represented the means of each group, and the lines on the bars indicated the standard deviations. Statistical significance was determined using a one-way ANOVA followed by post hoc Dunnett's test. ***p < 0.001 NIH, National Institutes of Health; AMP, antimicrobial peptides; ANOVA, analysis of variance; Ct, control; PB2, flatfish pardaxin; PIS, striped bass piscidin 1; TP4, tilapia piscidin 4

We have previously found that GC forms microcolonies and reduces the effects of antibiotics [24]. Hence, preventing microcolony formation can maximize the treatment efficacy. Since PB2, PIS, and TP4 inhibited GC growth, we sought to examine whether AMP can reduce microcolony formation. MS11 was inoculated into an eight-well chamber slide in the absence or presence of 10 μg/mL AMP and incubated for four hours, with azithromycin as positive control. The microcolony formation was visualized with a light microscope. Compared to the non-treatment control (Figure 4A), there were no apparent microcolonies, but small and loose GC aggregates in the AMP-treated group were found (Figure 4B-4D). Notably, GC incubated with PIS displayed nearly none of the microcolony formation (Figure 4C). To determine the reduction rate in microcolony, the size of each microcolony/aggregate was quantified (Figure 5A). We found that the size of the microcolony was reduced at least 4.5-fold in the AMP-treated group compared to non-treated GC (Figure 5B). These results suggest that PB2, PIS, and TP4 can reduce the microcolony formation of GC, thus potentially preventing GC biofilm formation.

PB2, PIS, and TP4 reduced GC adherence to endocervical epithelial cells

**Figure 6:**
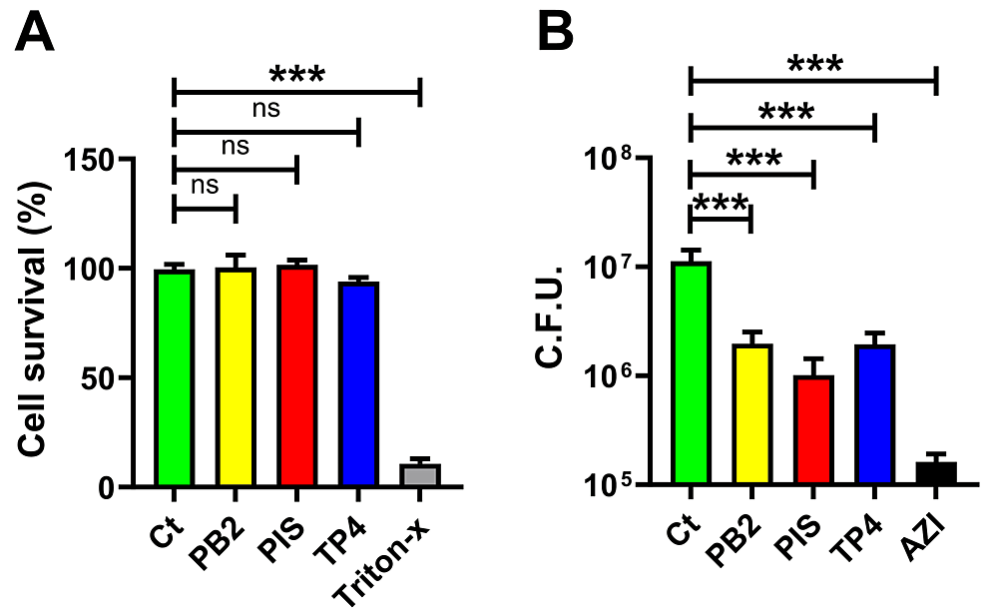
Effects of PB2, PIS, and TP4 on GC adherence onto ME180 endocervical epithelial cell. ME180 cells were grown on a 96-well plate, and 10 μg/mL of designated AMP was added with or without inoculating MS11. (A) To measure cell cytotoxicity, cells with or without AMP or 0.1% Triton-X were incubated for 24 hours, and CCK-8 assay was performed to determine the cell survival rate. (B) To evaluate GC adherence, cells with MS11 were incubated for three hours to determine the adherent GC. GC treated with 0.5 μg/mL of azithromycin (AZI) served as a positive control. Non-treated GC was served as control (Ct). The bars represented the means of each group with standard deviations. Statistical significance was determined using a one-way ANOVA followed by post hoc Dunnett's test. All data were collected from three independent experiments. ***p < 0.001 AMP, antimicrobial peptides; ANOVA, analysis of variance; CCK-8, Cell Counting Kit-8; ns, not significant; CFU, colony-forming units; PB2, flatfish pardaxin; PIS, striped bass piscidin 1; TP4, tilapia piscidin 4

GC colonization and infection only start after the GC's adherence to epithelial cells in the reproductive tract. Thus, adherence plays a crucial role in the initial colonization of GC on mucosal surfaces. Investigating whether PB2, PIS, and TP4 could inhibit GC adherence thereby lays clinical potential. Since these AMP may also affect epithelial cell viability, examining the effects of three AMP on both epithelial cells, and GC adherence is needed. To examine the effects of AMP on epithelial cells, ME180 cells were incubated with or without 10 μg/mL AMP for 24 hours, and the CCK-8 cell viability assay was conducted. Triton-X, a membrane-disrupting agent at a final concentration of 0.01%, was administered as a positive control. No significant difference in cell survival was found in cells treated with PB2, PIS, or TP4 compared to non-treatment control, while a significant 88% decrease was observed in the Triton-X group (Figure 6A). These results indicate that PB2, PIS, and TP4 have no effects on endocervical epithelial cells at concentrations above MIC in GC, thus confirming the usage of these AMP in examining their effects on GC adherence.

To assess the effects of AMP on adherence, MS11 was inoculated onto ME180 cells in the presence or absence of AMP for three hours. The cells were then collected and plated on GCK agar. Adherent GC were determined by colony counts. Azithromycin was used as a control for comparison. Compared to the non-treatment control, the AMP-treated groups were found to have a significant 6-10-fold reduction of adherent GC (p < 0.001, Figure 6B). Adherent GC in PIS treatment, though not significantly, were lower in number than those in PB2 and TP4. Nevertheless, the azithromycin treatment exhibited a significantly higher reduction in adherent GC cells compared to the AMP-treated group (p < 001). In conclusion, these data suggested that PB2, PIS, and TP4 can inhibit GC adherence, indicating colonization prevention.

## Discussion

*Neisseria gonorrhoeae* has become an emerging disease with its increased antibiotic resistance. Finding an alternative or supportive treatment is needed to prevent the transmission. Among the alternatives, antimicrobial peptides are easy to generate and have shown promising antibacterial activities. Teleost fish are one of the resources of antimicrobial peptides. Thus, this study evaluated the effect of major teleost-derived antimicrobial peptides against GC to assess their potential as prevention or treatment alternatives. Among the five AMP categorized into three families, piscidin-based PIS and TP4 and pardaxin-based PB2 had the lowest MIC against GC by 5-7.5 μg/mL. The TEM micrographs showed that the three AMP induced membrane deformation and the loss of cytoplasmic contents. Furthermore, these AMP could also reduce GC microcolony formation and adherence to endocervical epithelial cells, suggesting their clinical potential. Overall, this study provided a preliminary evaluation of teleost-derived AMP and their potential as an effective antibiotic alternative for future prevention/treatment development.

Antimicrobial peptides inhibit microorganisms differently based on families of AMP and the surface property of the specific microorganism. In this study, we found that hepcidin-based TH1-5 and piscidin-based TP3 had no or low inhibitory effect on GC, whereas piscidin-based PIS and TP4, as well as pardaxin-based PB2, had significant inhibitory effect in the tested concentration range. We reason that the results were attributed to two significant factors: the type and net charge of AMP. Pardaxin, with the property of amino acid sequences, has a relatively low net charge and therefore does not belong to the cationic peptide category. However, antimicrobial activities were found against many gram-positive and gram-negative bacteria at the AMP concentration range this study used [20]. Bhunia et al. found that pardaxin can bind to lipopolysaccharide (LPS) as one of the potential inhibitory mechanisms [25]. Thus, we speculate that a similar mechanism may occur in the lipooligosaccharide of GC. Hepcidin did not work against GC within the test range in this study.

In the previous study testing TH1-5, the authors also found that only a high concentration of 100 μg/mL or above could exhibit antibacterial activity against *Listeria monocytogenes* but not *Vibrio* spp. [26], indicating the low efficacy of TH1-5. Piscidins were reported widely as the broadest teleost-derived AMP against several gram-positive and gram-negative species [27]. Accordingly, different isoforms showed various MIC ranges against specific bacteria [28]. Our study found that only PIS and TP4 (piscidin-1 and piscidin-4) but not TP3 (piscidin-3) showed significant inhibition of GC in the tested range. We reason that both PIS and TP4 had higher isoelectric point (pI) (Table 1), thus giving them high cationic properties for interacting with GC as GC has a highly negatively charged outer membrane [29]. PIS has been shown to possess the most potent antimicrobial activity among all other piscidins [17]. Compared to PIS, TP3 and TP4 were reported to have less antimicrobial efficacy [28]. However, the TP4 used in this study originated from tilapia and was added with five arginine repeats [21], suggesting the enhanced binding to the GC surface over TP3 in alignment with PIS. Moreover, a similar observation by Zairi et al. examining amphibian-derived dermaseptin and its derivatives also showed that the increase of positively charged amino acid could increase the efficacy of AMP against GC by reducing MIC 10-fold [15]. Future work can involve the amino acid replacement for positively charged residues into these AMP to examine if increased efficacy can be obtained.

Many human cells showed different susceptibility from these AMP. Pardaxin has been shown to inhibit HeLa but not HT1080 cells at 15 μg/mL or more [30]. Piscidins also showed antitumor activity against HeLa and HT1080 cells at 20-25 μg/mL or more [31]. Our study used 10 μg/mL of these AMP as our working concentration and found no effect on the ME180 endocervical epithelial cells used in the infection model, suggesting the minimal working concentration for GC in the clinical setting. We found that the adherence GC was reduced under the treatment of the three AMP, yet the reduction level does not match the time-kill curve, whereas GC was mostly cleared. Two explanations are proposed: first, these AMP may neutralize the electronic charges. The reduction of GC surface charge has been shown to help GC adherence to the epithelial cells [29]. Positively charged PIS and TP4 may neutralize the electronic charge on the GC surface, thus potentially reducing the adherence to epithelial cells. Second, AMP can be absorbed by epithelial cells, thus reducing AMP's effects. Travkova et al. have shown that AMP can be absorbed by epithelial cells, which may affect the final concentration of AMP due to the interaction and binding of AMP to epithelial and bacterial cells [32]. It would be essential to examine the cytotoxicity of other associated reproductive epithelial cells in vitro or in vivo to determine the final concentration for clinical usage.

A retrospective study has summarized that the higher MIC of AMP may exist in resistant strains [33], implying strain specificity in susceptibility. Consistent with our study, we found that the azithromycin-resistant WHO Z strain, compared to MS11, has a higher MIC in PB2, PIS, and TP4. We hypothesized that the efflux pump may contribute to the elevated MIC. GC has an efflux pump system that can export antimicrobials such as antimicrobial peptides from the cytoplasm and periplasmic space. Such a system is mediated by the MtrCDE operon, which encodes major functional proteins for efflux pump [34]. The WHO Z strain has azithromycin resistance due to its mutation in an efflux pump-based MtrCDE system, increasing the efficiency of pumping out antimicrobials [35]. Studies using mice have shown that the mutation can also enhance the fitness of gonococci during the experimental infection of the lower genital tract of female mice [36], which supports the concept that the MtrCDE efflux pump is of importance to export host-derived antimicrobials such as cationic AMP. Therefore, these teleost-derived AMP, like others, have restrictions in efflux pump-based antibiotic resistance in GC. Future work will involve structural modification or adding efflux pump inhibitors, such as peptides targeting MtrCDE [16] for potential clinical usage.

Our previous work has evaluated the effects of a tellurium-based inorganic compound (AS101) on GC [37]. Comparing teleost-derived AMP to AS101, we found that the teleost-derived AMP possess more bactericidal properties than AS101, as shown in the time-kill curve and TEM, thus suggesting that less antibiotic resistance could occur using AMP. Nevertheless, AMP may not be as effective in the azithromycin-resistant strain as in the nonresistant strain, whereas AS101 showed similar inhibition between both strains. Nevertheless, AMP and AS101 reduce GC microcolony formation and colonization on epithelial cells. Therefore, AMP and AS101, two antimicrobials, may work in different clinical settings to treat GC infection.

Although this study explored the efficacy of teleost-derived AMP in GC, it has limitations. First, assays in this study were performed in an in vitro environment, thus lacking evidence of GC inhibition in the actual reproductive organs. Besides, the female reproductive tract harbors a unique niche of microbiota to maintain low pH in the vaginal region. How these AMP inhibit GC while interacting with microbiota under low pH will be of clinical importance. Second, the time-kill assay and TEM allow us to conclude AMP's bactericidal property in inducing GC membrane damage and the loss of cytoplasmic contents. However, whether these damaging events were dependent or independent of each other was not known. It would be essential to examine if these AMP possess multiple GC inhibitory functions by investigating the membrane blebbing, breakage, and the loss of cell contents in a time-dependent manner.

## Conclusions

The present study shows that pardaxin- and piscidin-based AMP derived from teleost fish are effective against pathogenic *Neisseria gonorrhoeae* at a concentration nontoxic to epithelial cells. Therefore, these AMP against more recent clinical isolates can be evaluated for developing a clinical prevention and treatment. For the future development of these AMP against reproductive pathogens, one can combine AMP and existing antibiotics for examining synergistic inhibition and may also assess the inhibitory effects on other harmful bacteria in the reproductive tract, such as *Chlamydia*, *Gardnerella*, and *Mycoplasma*. The teleost-derived AMP thereby may individually serve or be combined with antibiotics as the potential medical regimens for not only sexually transmitted infections but also overall reproductive health.
